# Dimensions of cognitive reserve and their predictive power of cognitive performance and decline in the elderly

**DOI:** 10.3389/frdem.2023.1099059

**Published:** 2023-08-31

**Authors:** Teodoro del Ser, Elizabeth Valeriano-Lorenzo, Luis Jáñez-Escalada, Marina Ávila-Villanueva, Belén Frades, María-Ascensión Zea, Meritxell Valentí, Linda Zhang, Miguel A. Fernández-Blázquez

**Affiliations:** ^1^Clinical Department, Alzheimer's Center Reina Sofia—CIEN Foundation, Madrid, Spain; ^2^Institute of Knowledge Technology, Complutense University, Madrid, Spain; ^3^Neuroimaging Department, Alzheimer's Center Reina Sofia—CIEN Foundation, Madrid, Spain

**Keywords:** cognitive reserve, dimensions, cognitive performance, mild cognitive impairment, brain volume, brain atrophy

## Abstract

**Background:**

The relative importance of different components of cognitive reserve (CR), as well as their differences by gender, are poorly established.

**Objective:**

To explore several dimensions of CR, their differences by gender, and their effects on cognitive performance and trajectory in a cohort of older people without relevant psychiatric, neurologic, or systemic conditions.

**Methods:**

Twenty-one variables related to the education, occupation, social activities, and life habits of 1,093 home-dwelling and cognitively healthy individuals, between 68 and 86 years old, were explored using factorial analyses to delineate several dimensions of CR. These dimensions were contrasted with baseline cognitive performance, follow-up over 5 years of participants' cognitive trajectory, conversion to mild cognitive impairment (MCI), and brain volumes using regression and growth curve models, controlling for gender, age, marital status, number of medications, trait anxiety, depression, and ApoE genotype.

**Results:**

Five highly intercorrelated dimensions of CR were identified, with some differences in their structure and effects based on gender. Three of them, education/occupation, midlife cognitive activities, and leisure activities, were significantly associated with late-life cognitive performance, accounting for more than 20% of its variance. The education/occupation had positive effect on the rate of cognitive decline during the 5-year follow up in individuals with final diagnosis of MCI but showed a reduced risk for MCI in men. None of these dimensions showed significant relationships with gray or white matter volumes.

**Conclusion:**

Proxy markers of CR can be represented by five interrelated dimensions. Education/occupation, midlife cognitive activities, and leisure activities are associated with better cognitive performance in old age and provide a buffer against cognitive impairment. Education/occupation may delay the clinical onset of MCI and is also associated with the rate of change in cognitive performance.

## Introduction

Epidemiological, clinical, neuropsychological, and neuroimaging studies have shown that cognitive training and other social and mentally stimulating activities have protective effects against the clinical and cognitive manifestations of neural injuries or neurodegeneration (Ikanga et al., 2017; Stern et al., 2018; Nunes and Silva Nunes, 2021). Cognitive reserve (CR) is a theoretical construct proposed to account for those observations in clinical, epidemiological (Stern et al., 2018), and cognitively healthy (CH) cohorts (Opdebeeck et al., 2016). Individuals with higher proxy markers of CR demonstrate better cognitive performance and lower rates of prevalent and incident dementia (Pettigrew and Soldan, 2019).

There is a lack of consensus on the best measure of CR construct (Harrison et al., 2015; Nogueira et al., 2022). It is usually operationalized as education level (O'Shea et al., 2015), but other variables, such as occupation attainment (Hakiki et al., 2021); reading level (O'Shea et al., 2015); intelligence quotient (Whalley et al., 2015), engagement in cognitively stimulating activities (Opdebeeck et al., 2016), a composite of education, labor, leisure, and social activities (Harrison et al., 2015); or a questionnaire designed for this purpose (Nucci et al., 2012; Kartschmit et al., 2019), are also used as proxies of CR. CR is assumed to accumulate over a life span (Xu et al., 2019; Song et al., 2022) with different components (Rouillard et al., 2017), but the relative importance of the effects of each one on cognitive performance and prevention or delay of late decline has not been comprehensively tested (Rouillard et al., 2017). The relationships between the level of CR and longitudinal cognitive trajectories and outcomes in CH people, as well as its structural brain substrates, are also unclear (Pettigrew and Soldan, 2019).

This study has three main objectives: the first one is to identify the internal structure of CR and quantify its different dimensions by means of a multidimensional analysis of a set of cognitive, social, and leisure activities undertaken during childhood and adulthood by a cohort of CH older people. The second one is to examine in the longitudinal assessment of this cohort the associations of these CR dimensions with a baseline cognitive performance, the clinical status over a 5-year follow-up, and the cognitive trajectory of individuals with persistent cognitive normality and those who converted to mild cognitive impairment (MCI). Finally, the associations of these CR dimensions with some measures of brain volume, as proxy markers of brain reserve, have been also ascertained (Supplementary Table 1). Our analyses were guided by three hypotheses: (a) several different dimensions can be disclosed in the CR construct; (b) CR dimensions have a positive but different effect on cognitive performance, cognitive decline, and cognitive trajectory; and (c) CR may have a positive effect on brain volume (Supplementary Table 1). Since the components of these CR dimensions, as well as cognitive trajectories in older cohorts (Levine et al., 2021) and brain volumes show differences between men and women, the analyses have also been performed by gender.

## Methods

### Setting and subjects

**Figure 1:**
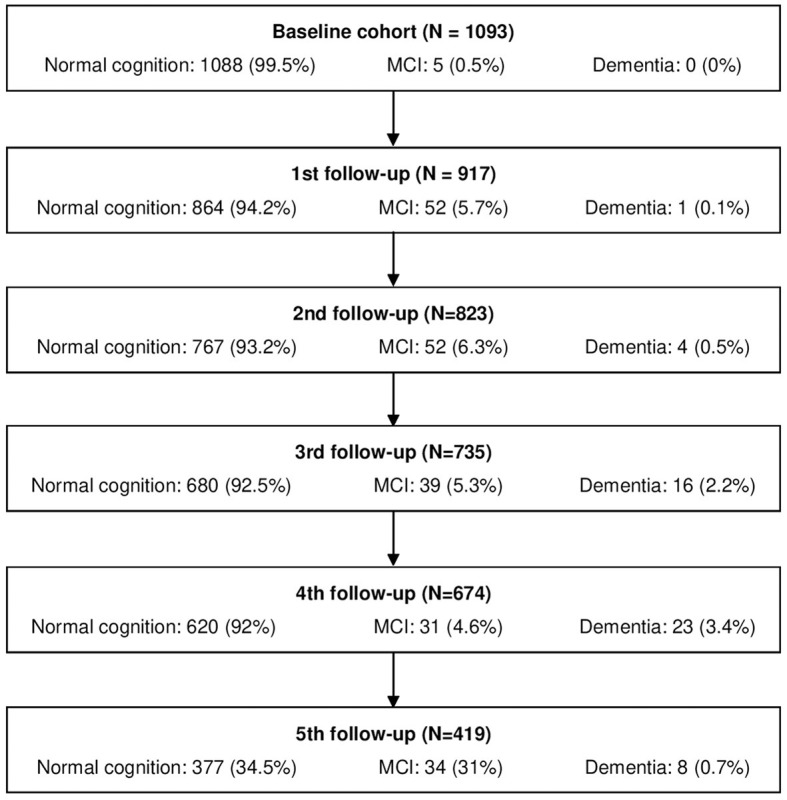
Individuals in the survey at every yearly assessment. MCI, mild cognitive impairment.

The Vallecas Project is an ongoing single-center, multidisciplinary, observational, longitudinal cohort study planned to identify early markers of cognitive impairment in the elderly (Olazarán et al., 2015). The participants are home-dwelling volunteers aged 68 to 86 years at baseline, without previous relevant psychiatric (schizophrenia, bipolar disorder, major depression), neurologic (stroke with cognitive or motor sequelae, head trauma with loss of consciousness, structural brain lesions, epilepsy, brain infections, mental retardation), or systemic (active carcinoma, alcohol or drug abuse, nutritional deficiencies) disorders. They were recruited between 2011 and 2013 through radio, TV, and leaflet campaigns and through visits to centers for the elderly in Madrid, Spain. After giving informed consent, they undertook annual systematic assessments (Figure 1) including sociodemographic data, family history of neurological and psychiatric diseases, medical history, lifestyle habits, neurological and neuropsychological exams, blood collection, and brain magnetic resonance imaging (MRI) scans. The Vallecas Project was approved by the Ethics Committee of the Carlos III Health Institute.

A total of 2,077 individuals were screened, of which 1,169 were finally included in the Vallecas Project database. This study has been performed with the data obtained at baseline and in five follow-up assessments of 1,093 participants (702 women, 64.2%) who provided the selected variables, accounting for 4,235 person-year assessments and aged 74.1 ± 3.9 years at baseline.

### Variables

Five sets of variables were selected from the Vallecas Project database:

*Reserve-related variables* according to the current definition of CR (Harrison et al., 2015; Ikanga et al., 2017; Stern et al., 2018) recorded at baseline.

**(a) Education:** education level (less than primary school, primary school, high school, more than high school), number of languages spoken (Spanish; two languages from Spain; Spanish and one foreign language; three or more languages), additional professional education in adulthood (none; 1–2 courses; 2–5 courses; >5 courses)

**(b) Occupation:** occupation level (not qualified, manual worker, qualified worker, professional, manager) and years worked

**(c) Life habits in midlife (30–65 years):** A questionnaire was designed for the purposes of this project using the Lifetime of Experiences Questionnaire (Valenzuela and Sachdev, 2007) as a model. Thus, 16 items referring to everyday activities that require or provide cognitive experience were selected (artistic activities, social activities, light physical exercise, moderate physical exercise, news and newspapers, electronic devices, music listening, reading, writing, traveling, hobbies, card games, intellectual games, collecting, attending lectures, and attending shows). Participants scored every item on a 5-point scale (0: never; 1: less than once a month; 2: every month; 3: every 2 weeks; 4: every week). Of these life habits, 1–30% were missing data for several variables. Because the missing data were not randomly missing, multiple imputations were obtained based on the maximal likelihood multiple imputation method. No data were available about the life habits during the participants' young adulthood.

All the CR-related quantitative variables were standardized, and the ordinal variables were treated with the conditional median scoring approach (Chen and Wang, 2014) to obtain *z*-scores and better fit the requirements of factor analyses.

#### Cognitive assessments

An extensive neuropsychological battery was administered at every visit to the Vallecas Project (Olazarán et al., 2015). We selected for this study a subset of cognitive parameters that track most of the cognitive spectrum and are associated with a higher risk of conversion to MCI (Fernández-Blázquez et al., 2016; Del Ser et al., 2019): general cognitive status [Mini Mental State Examination [MMSE] (Folstein et al., 1975)], episodic memory [total immediate and delayed recall in the Free and Cued Selective Reminding Test (Buschke, 1984)], language (fluency of animals in 1 min), visuo-constructive ability [clock drawing test (Cacho et al., 1999)], and attention and executive functioning [digit-symbol test (Wechsler, 1997)]. The scores in those tests recorded at baseline and at five annual assessments were taken for analyses. The raw scores, excluding MMSE, were transformed to *z*-scores and averaged to obtain a global *z*-composite cognitive score for every individual at every assessment. To handle data sets with omissions due to the attrition of the cohort, cognitive data lost during the follow-up were obtained by multiple imputations based on the predictive mean matching method, especially recommended for non-normally distributed variables, using 50 imputed data pools.

#### Clinical diagnosis

The cognitive status of every participant was diagnosed at baseline and at every follow-up visit with a consensus between the neurologist and the neuropsychologist, taking into account their age, functional status, cognitive performance (Olazarán et al., 2015), and brain MRIs indicating CH, MCI, or dementia. The criteria from the National Institute on Aging-Alzheimer's Association (Albert et al., 2011) and from the *Diagnostic and Statistical Manual of Mental Disorders*, fourth edition, text revision (Olazarán et al., 2015), were used to diagnose MCI and dementia, respectively. The age at the time of the MCI diagnosis was established for every individual.

#### Groups

The individuals were divided into two groups according to their diagnosis during the follow-up: those who were CH throughout the follow-up period and those who converted to MCI at any time during the follow-up period.

#### Brain volumes and atrophy

At baseline (Olazarán et al., 2015), 974 3T MRI brain scans were available from 974 participants. A T1-weighted image for every subject was segmented into gray matter, white matter, and cerebrospinal fluid (CSF) volumes using SPM12 software (http://www.fil.ion.ucl.ac.uk/spm/, version 6225), and total intracranial volume (TIV) was obtained by summing the three volumes. The percentage of TIV represented by CSF was calculated as a proxy of brain atrophy, and the gray and white matter volumes were corrected by TIV.

#### Covariates

ApoE genotypes [rs429358 and rs7412, determined by real-time polymerase chain reaction (Calero et al., 2009)], gender, and age, as well as the number of medications taken, trait anxiety [State-Trait Anxiety Inventory (Spielberger et al., 1970)], marital status (non-married [single, divorced, or widowed] vs. married), and depressive symptoms [Geriatric Depression Scale (Sheikh and Yesavage, 1986)] at baseline, were used as covariates. Brain atrophy was also a covariate to better assess the effects of CR dimensions, separating out their interactions with aged brain status (Stern et al., 2018).

### Statistical analyses

#### Internal structure of CR

A factor analysis of 21 variables was performed to determine the underlying dimensions of CR. The 1,093 cases from the sample were randomly separated into two groups: an exploratory factor analysis (EFA) was performed for one group and a confirmatory factor analysis (CFA) was performed for the other group as a cross-validation procedure (Anderson and Gerbing, 1988) of the model. Multivariate normality (*p* < 0.05), kurtosis, and asymmetry tests (indices > 2) were previously checked.

#### EFA

The unweighted least squares for factorial estimation and the oblique rotation solution were used. The best model was selected according to the following criteria: (1) communality of the variables; (2) goodness of fit according to the chi-square difference test (Δχ^2^), Tucker–Lewis index (TLI; ≥0.95), and root mean square error of approximation (RMSEA; ≤ 0.05); (3) average variance extracted for the model; (4) factor loadings; and (5) correspondence of the obtained factors with previous literature.

#### CFA

The best model selected from the EFA was tested using CFA. Every observed variable was fixed to its respective latent dimension following the structure of CR explored with the EFA. The factor loadings and the correlations between the latent variables were estimated. The weighted least square means and the adjusted variance were used as methods for estimation. The average variance extracted and the construct reliability were analyzed.

#### Multigroup invariance analysis

In the final structure of CR, with five latent dimensions, the whole sample was tested with a multigroup invariance analysis by gender to determine if the model of CR was measured consistently in both groups. Configural, weak metric, and partial-weak metric invariances, as nested models, were compared to test the hypotheses that the number of factors (configural invariance), and the factor loadings (weak metric invariance) were similar across groups. The Δχ^2^ and the difference of CFI of < 0.01 were used to compare the two models (Vandenberg and Lance, 2000).

#### Relationships of CR dimensions

##### Association of CR dimensions with cognitive performance

Multiple regression analyses were performed with every cognitive test score as the dependent variable and the factorial scores in the CR dimensions as independent predictors. These analyses were performed for the sample of 973 individuals (334 men, 631 women) who had factorial scores in the CR dimensions and had provided data for all the cognitive variables and the nine covariates.

##### Association of cognitive reserve dimensions with MCI

Cox regression analyses were performed with those who converted to MCI during the 5-year follow-up as a dependent variable. Time to event was the time from baseline until the exam when the diagnosis of MCI was made or until the last exam in persistently CH cases who were censored by the date of their last assessment. These analyses were performed for 843 individuals (300 men, 543 women) who were followed up for 4.24 ± 1.5 years with at least one follow-up visit, including 94 (37 men, 57 women) who had converted to MCI.

In all these analyses, the dimensions of CR, the demographic covariates, and brain atrophy were introduced in independent blocks to reduce the effect of collinearity and multiple testing. The regression analyses were performed separately for every gender group, using the factorial scores of the CR dimensions in the corresponding factorial model and for the whole group, introducing gender as a covariate.

#### Association of CR dimensions with cognitive trajectory

**Figure 2:**
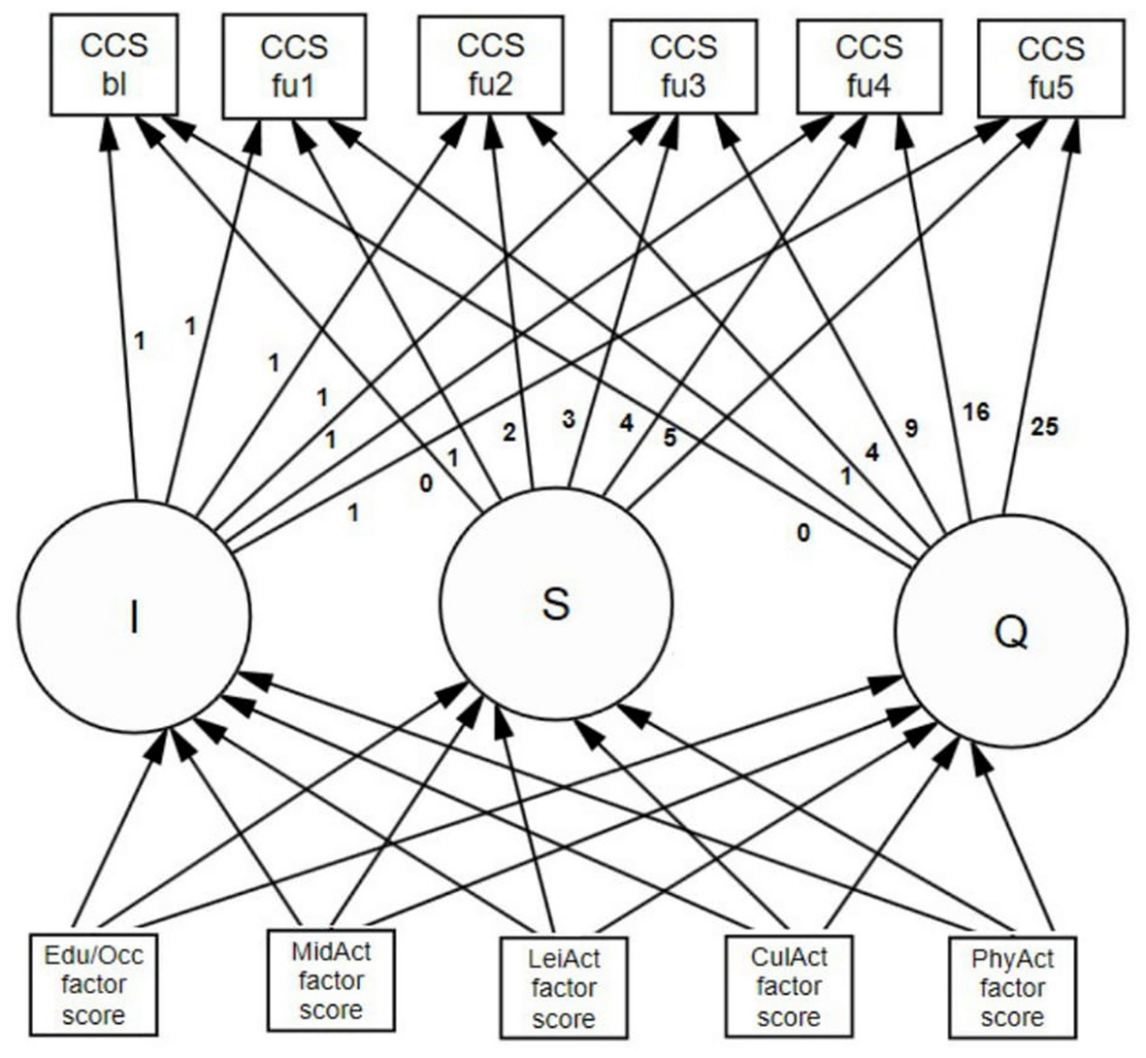
Path diagram of conditional latent growth model fitted to cognitive reserve, adjusted by relevant covariates. ^1^Five dimensions of Cognitive reserve: education/occupation, midlife cognitive activities, leisure activities, cultural activities, and physical activities. Nine covariates such as age at baseline, marital status, number of medications, trait anxiety and depression scores at baseline, gender, number of ApoE-ε4 and ApoE-ε2 alleles, and atrophy. I, intercept; S, linear slope; Q, quadratic slope; CCS, cognitive composite score; bl, score at baseline; fu1, score at first follow-up; fu2, score at second follow-up; fu3, score at third follow-up; fu4, score at fourth follow-up; fu5, score at fifth follow-up. ^1^This diagram represents the latent growth curve model used for the group of individuals diagnosed with mild cognitive impairment. The model used in the group of cognitively healthy cases did not include the quadratic slope.

Latent growth curve models (LGCMs) of the composite *z*-scores along the five follow-up assessments were performed in a sample of 973 subjects according to the structural equation modeling framework. First, a multiple indicators multiple causes model was performed to examine the effect of the diagnosis on the cognitive trajectory. It was expected that CH subjects and MCI converters had different baseline cognitive levels and rates of change across time. Second, linear and quadratic unconditional models by the cognitive-diagnosis group were compared to find the best model fit. Since these models were nested within each other, Δχ^2^ was used to select the best model. Finally, a conditional LGCM was performed (see Figure 2). It tested the influence of the CR dimensions and covariates on the growth factors of each diagnostic group (CH and MCI). The covariates considered in the analysis were age, marital status, number of medications, trait anxiety, depression, brain atrophy at baseline, gender, and number of ApoE-ε4 and ApoE-ε2 alleles.

#### Association of CR dimensions with brain volumes

Similar multiple regression analyses were performed by gender, using gray matter and total volumes corrected by TIV as dependent variables and the same covariates.

R program (version 4.0.3) was employed for the factor analyses, using libraries such as psych version 2.2–5 (Revelle, 2022), lavaan version 0.6–8 (Rosseel, 2012), sem version 3.1–11 package (Fox et al., 2020), to address multiple imputations of missing data; mice version 3.13.0 (van Buuren and Groothuis-Oudshoorn, 2011), to perform the growth curve modeling; and semTools version 0.5–6 (Jorgensen et al., 2022) and SPSS (version 20), for the regression and Cox analyses. The standardized coefficients, βs, of variables and the adjusted coefficients of determination (*R*^2^) as measures of size effect are presented to respectively capture the relative effect and the explained variance of CR dimensions and covariates in the regression analyses.

## Results

The demographic and clinical data of participants at baseline are summarized in Table 1. After a follow-up of 4.2 ± 1.5 years (range: 0.97–6.75 years), 112 participants (10.3%) were diagnosed with MCI at the age of 78.4 ± 4.1 years, and 31 of them (2.8%) progressed to dementia (Table 1 and Figure 1); 20 additional individuals had a diagnosis of MCI but reverted to healthy cognition before the last assessment and were considered to have cognitively normal health for the longitudinal analyses.

**Table 1:** Baseline features of the cohort.

	**Total sample (*N* = 1,093)**	**Men (*n* = 391)**	**Women (*n* = 702)**
	**Mean (SD) [range]**	**Mean (SD) [range]**	**Mean (SD) [range]**
Age at baseline (years)	74.1 (3.9) [68–86]	74.1 (3.9) [68–86]	74.2 (3.9) [68–86]
Education (years)^***^	10.6 (5.8) [0–24]	12.1 (6.2) [0–24]	9.7 (5.4) [0–24]
Years worked^***^	27 (19.2) [0–60]	43.4 (8.5) [3–60]	17.8 (17.3) [0–60]
Number of medications^a^	3.4 (2.3) [0–13]	3.3 (2.3) [0–13]	3.4 (2.3) [0–13]
Mini mental state examination	28.6 (1.5) [20–30]	28.7 (1.4) [20–30]	28.6 (1.6) [20–30]
FCSRT immediate recall^*^	23.6 (6.2) [7–44]	23.1 (6.2) [7–44]	23.9 (6.2) [7–44]
FCSRT delayed recall	9.4 (2.6) [0–16]	9.3 (2.6) [0–16]	9.6 (2.7) [0–16]
Word fluency^**^	18.5 (8.7) [5–34]	19.1 (4.9) [5–34]	18.3 (4.8) [5–34]
Clock drawing test^***^	9.4 (1.2) [4–10]	9.6 (0.9) [4–10]	9.2 (1.2) [4–10]
Digit symbol test^***^	19.3 (7.4) [1–43]	21 (7.6) [1–43]	18.5 (7) [1–43]
Geriatric depression scale^***^	1.5 (2.19) [0–14]	1 (1.6) [0–14]	1.9 (2.5) [0–14]
Trait anxiety (STAI)^***^	16.9 (9.58) [0–49]	13.9 (8.6) [0–49]	18.7 (10) [0–49]
White matter volume (mL)^b^	534 (40) [356–723]	535 (40) [356–723]	533 (39) [356–723]
Gray matter volume (mL)^b***^	815 (57) [613–963]	785 (54) [613–963]	830 (52) [613–963]
Brain atrophy (%)^b, c***^	31 (4) [17–44]	32.1 (3.7) [17–44]	30.6 (4.3) [17–44]
	***n*** **(%)**	***n*** **(%)**	***n*** **(%)**
**Education** ^***^			
< Primary school	205 (18.8)	57 (14.6)	148 (21.1)
Primary school	328 (30.0)	84 (21.5)	244 (34.8)
High school	269 (24.6)	107 (27.4)	162 (23.1)
>High school	291 (26.6)	143 (36.6)	145 (21.1)
**Occupation** ^d***^			
Not qualified	298 (27.3)	48 (14)	250 (43.9)
Manual worker	207 (18.9)	94 (27.4)	113 (19.8)
Qualified worker	143 (13.1)	49 (14.3)	94 (16.5)
Professional	207 (18.9)	100 (28.2)	107 (16.8)
Manager	58 (5.3)	52 (15.2)	6 (1.1)
**ApoE genotype** ^e^			
ε4 heterozygous	182 (17.1)	74(19.1)	112 (16)
ε4 homozygous	5 (0.5)	1 (0.3)	4 (0.6)
ε2 heterozygous	138 (12.7)	58 (14.9)	80 (11.4)
ε2 homozygous	1 (0.1)	1 (0.3)	0 (0)
**Assessments**			
Baseline	1,093 (100)	391 (100)	702 (100)
1st follow-up	917 (83.9)	336 (86)	569 (81.1)
2nd follow-up	823 (75.3)	308 (78.8)	512 (72.9)
3rd follow-up	735 (67)	276 (70.6)	449 (64)
4th follow-up	674 (61.7)	251 (64.2)	414 (59)
5th follow-up	419 (38.3)	169 (43.2)	250 (35.6)
**Diagnosis during follow-up**			
MCI^f^	112(10.3)	44 (11.3)	68 (9.7)
Dementia	31 (2.8)	7 (1.8)	24 (3.4)

MCI, mild cognitive impairment; STAI, State-Trait Anxiety Inventory; FCSRT, Free and Cued Selective Reminding Test.

^a^Data available in 1,072 cases.

^b^Data available in 974 cases.

^c^Brain atrophy: percentage of the total intracranial volume represented by cerebral spinal fluid volume.

^d^Data available in 913 cases.

^e^Data available in 1,088 cases. Only data on e4 and e2 genotypes are presented.

^f^An additional 20 individuals were diagnosed with MCI but reverted to healthy cognition.

^*^p < 0.05; ^**^p < 0.01; ^***^p < 0.001.

Women had significantly lower education levels, occupation attainment, and time at work; lower scores in word fluency, the clock drawing test, and the digit symbol test; more gray matter volume and less brain atrophy; and higher scores for immediate memory, depressive symptoms, and trait anxiety (Table 1).

### Internal structure of CR

#### EFA

An EFA was conducted with a subset of 543 subjects. The preliminary evaluations about its adequacy showed an measure of sampling adequacy of 0.89 on the Kaiser–Meyer–Olkin test and a significant Bartlett's test of sphericity (*p* < 0.0001). The first parallel analysis suggested five factors with observed eigenvalues greater than estimated eigenvalues. Then, factor solutions with oblimin rotation were analyzed for three models with four, five, and six factors (Supplementary Table 2). The model of five factors was selected because it explained 47% of the total variance and showed acceptable goodness of fit indices (TLI = 0.932, RMSEA = 0.05, CI 90% [0.04, 0.06]), communalities between 0.18 and 0.84, and a clearer structure of the factor loadings of the observed variables.

#### CFA

A structure of five CR dimensions from 21 observed variables was analyzed in the second randomly split sample of 550 subjects. The goodness of fit indices showed very good values: CFI = 0.96, TLI = 0.98, RMSEA = 0.045, CI 90% [0.041, 0.049], and standardized root mean square residual = 0.047, indicating a good fit between the model and the observed data. The omega index, between 0.61 and 0.83, indicates a high reliability of the factors. The factor loadings were also higher with values ranging from 0.32 to 0.88.

#### Multigroup invariance analysis

The structure of five CR dimensions obtained in the CFA was tested for multigroup invariance by gender. The Δχ^2^ and the difference in the CFI index demonstrated configurational invariance but not weak metric invariance. Two models of partial-weak metric invariance with three and five freed parameters were examined. After testing the four nested models, only configurational invariance was achieved (Supplementary Table 3), indicating that the structure of the five CR dimensions is the same for both genders, but the factor loadings are different between men and women.

Finally, the factor loadings (Tables 2a, b) and the factorial scores were obtained separately for men and women. According to the factor loadings, the five factors were labeled education/occupation, midlife cognitive activities, leisure activities, cultural activities, and physical activities. Each factor had an extracted variance from 29% to 52%. The five factors were significantly correlated between them in both models (*r* = 0.21–0.79; Tables 2a, b). The factor scores obtained for every individual were recorded and introduced in the following regression analyses.

**Table 2a:** Confirmatory factor analysis of 21 variables related to cognitive reserve in the sample of 702 women: factor loadings and correlations.

**Variables**	**Factors**				
	**Education/ occupation**	**Midlife cognitive activities**	**Leisure activities**	**Cultural activities**	**Physical activities**
Level of formal education	0.83				
Occupation level	0.81				
Education in the adulthood	0.82				
Spoken languages	0.50				
Years at work	0.53				
Attending shows		0.74			
Traveling		0.63			
Reading		0.64			
News and newspapers		0.63			
Music listening		0.51			
Electronic devices		0.65			
Attending lectures		0.71			
Intellectual games			0.71		
Hobbies			0.73		
Card games			0.44		
Artistic activities				0.53	
Writing				0.54	
Social activities				0.62	
Collecting				0.38	
Light exercise					0.70
Moderate exercise					0.69
Construct reliability (omega index)	0.83	0.83	0.66	0.61	0.65
Average variance extracted	52%	42%	41%	29%	48%
**Inter-correlation of factors**					
Education/occupation	1.00				
Midlife cognitive activities	0.74^***^	1.00			
Leisure activities	0.31^***^	0.44^***^	1.00		
Cultural activities	0.66^***^	0.79^***^	0.43^***^	1.00	
Physical activities	0.29^***^	0.55^***^	0.35^***^	0.43^***^	1.00

^***^p < 0.001.

**Table 2b:** Confirmatory factor analysis of 21 variables related to cognitive reserve in the sample of 391 men: factor loadings and correlations.

**Variables**	**Factors**				
	**Education/occupation**	**Midlife cognitive activities**	**Leisure activities**	**Cultural activities**	**Physical activities**
Level of formal education	0.82				
Occupation level	0.79				
Education in the adulthood	0.82				
Spoken languages	0.48				
Years at work	0.07				
Attending shows		0.65			
Traveling		0.61			
Reading		0.73			
News and newspapers		0.63			
Music listening		0.39			
Electronic devices		0.71			
Attending lectures		0.66			
Intellectual games			0.70		
Hobbies			0.88		
Card games			0.32		
Artistic activities				0.58	
Writing				0.71	
Social activities				0.50	
Collecting				0.47	
Light exercise					0.79
Moderate exercise					0.55
Construct reliability (omega index)	0.81	0.82	0.68	0.66	0.63
Average variance extracted	52%	40%	45%	33%	47%
**Inter-correlation of factors**					
Education/occupation	1.00				
Midlife cognitive activities	0.76^***^	1.00			
Leisure activities	0.27^***^	0.36^***^	1.00		
Cultural activities	0.63^***^	0.74^***^	0.37^***^	1.00	
Physical activities	0.27^***^	0.54^***^	0.21^**^	0.44^***^	1.00

^**^p < 0.01; ^***^p < 0.001.

### Relationships of CR dimensions

#### CR dimensions and cognitive performance at baseline

The multiple regression analyses performed on the cognitive data showed that the education/occupation dimension was significantly associated with almost all the cognitive test scores (β: 0.127–0.522, *p* < 0.001, for most of them; Table 3a) except the clock drawing test and immediate and delayed memory in men; the leisure activities dimension was associated in both genders with immediate memory (β: .096, *p* < 0.01), word fluency, digit symbol test, and the composite score (β: 0.151, 0.159, and 0.141, respectively; *p* < 0.001; Table 3a); the midlife cognitive activities dimension was associated with immediate memory in the whole group and with the digit symbol test in women (β: 0.177 and 0.184, respectively; *p* < 0.05; Table 3a); and physical activities was negatively associated with the digit symbol test in men (β: −0.119, *p* < 0.05).

**Table 3a:** Association of cognitive reserve dimensions with cognitive performance by gender.

	**Cognitive reserve dimensions**					**Explained variance**	
	**Education/ occupation**	**Midlife cognitive activities**	**Leisure activities**	**Cultural activities**	**Physical activities**	$R_{\text{CR}}^{2}$	$R_{\text{M}}^{2}$
**MMSE**							
Men	**0.295** ^**^	0.020	0.062	−0.041	0.037	0.090	0.112
Women	**0.323** ^***^	0.056	0.034	−0.052	0.021	0.113	0.128
Whole group	**0.302** ^***^	0.048	0.043	−0.053	0.027	0.110	0.129
**Clock drawing test**							
Men	−0.124	0.154	0.042	0.100	−0.086	0.002	0.012
Women	0.137	−0.043	0.042	0.059	0.043	0.039	0.046
Whole group	0.052	0.065	0.040	0.039	−0.001	0.027	0.046
**Immediate memory**							
Men	0.123	0.119	**0.128** ^*^	−0.045	−0.071	0.048	0.134
Women	**0.143** ^*^	0.191	0.077	−0.115	0.006	0.095	0.152
Whole group	**0.127** ^*^	**0.177** ^*^	**0.096** ^**^	−0.098	−0.018	0.081	0.149
**Delayed memory**							
Men	0.158	0.118	0.050	−0.117	0.012	0.034	0.077
Women	**0.232** ^**^	0.003	0.024	−0.002	0.014	0.079	0.130
Whole group	**0.203** ^***^	0.061	0.033	−0.063	0.016	0.066	0.120
**Word fluency**							
Men	**0.316** ^**^	−0.107	**0.223** ^***^	−0.007	0.041	0.130	0.181
Women	**0.193** ^**^	0.178	**0.119** ^**^	−0.109	−0.042	0.108	0.158
Whole group	**0.226** ^***^	0.063	**0.151** ^***^	−0.053	−0.008	0.115	0.173
**Digit-symbol test**							
Men	**0.522** ^***^	0.045	**0.229** ^***^	−0.031	−0.119^*^	0.371	0.421
Women	**0.456** ^***^	**0.184** ^*^	**0.117** ^**^	−0.132	−0.004	0.371	0.420
Whole group	**0.477** ^***^	0.123	**0.159** ^***^	−0.084	−0.050	0.355	0.431
**Composite score**							
Men	**0.330** ^**^	0.078	**0.210** ^***^	−0.036	−0.063	0.206	0.293
Women	**0.322** ^***^	0.144	**0.108** ^**^	−0.084	0.006	0.236	0.311
Whole group	**0.314** ^***^	0.139	**0.141** ^***^	−0.076	−0.018	0.226	0.310

Data are standardized coefficients, βs, and adjusted coefficients of determination of the multiple regression models ($R_{\text{CR}}^{2}$ for cognitive reserve and $R_{\text{M}}^{2}$ for the entire model). Significant coefficients are presented in bold characters. Included in the analysis were 334 men and 631 women.

MMSE, Mini Mental State Examination.

^*^p < .05; ^**^p < .01; ^***^p < .001.

Small but significant negative effects of gender were found in the clock drawing test, word fluency, digit symbol test, and composite score (β standardized coefficients: −0.119, −0.104, −0.169, and −0.092, respectively; Table 3b). Age and depressive symptoms were significantly and negatively associated with many cognitive scores for both genders (β: −0.106 to −0.218 and −0.078 to −0.225, respectively; Table 3b), whereas trait anxiety was positively associated (β: .067–.158; Table 3b) and the number of drugs was negatively associated (β: −0.081 to 0.115; Table 3b) with several cognitive scores, mainly in women. Brain atrophy showed a significant negative association with immediate memory and the digit symbol tests in men (β: −0.115 and –.112, *p* < 0.05; Table 3b) and with delayed memory, word fluency, and composite scores for both genders (β: −0.077, −0.096, and −0.081, respectively, *p* < 0.05; Table 3b).

**Table 3b:** Association of covariates with cognitive performance by gender.

	**Covariates**									**Explained variance**
	**Age**	**Female gender**	**Marital status**	**Depressive symptoms**	**Trait Anxiety**	**Number of drugs**	**ApoE-ε4 alleles**	**ApoE-ε2 alleles**	**Brain atrophy**	** $R_{\text{C}}^{2}$ **
**MMSE**										
Men	−0.067	–	−0.068	−0.157^*^	**0.119** ^*^	−0.073	−0.001	−0.050	−0.015	0.022
Women	−0.021	–	0.047	−0.151^**^	**0.158** ^**^	−0.007	−0.025	0.004	−0.043	0.015
Whole group	−0.040	−0.051	0.017	−0.150^***^	**0.145** ^***^	−0.031	−0.016	−0.014	−0.038	0.019
**Clock drawing test**										
Men	−0.071	–	−0.002	−0.002	0.054	−0.115^*^	−0.091	−0.057	−0.066	0.010
Women	−0.060	–	0.025	−0.040	0.010	−0.067	−0.051	0.061	−0.005	0.007
Whole group	−0.065	−0.119^**^	0.021	−0.043	0.025	−0.022	−0.060	0.024	−0.015	0.021
**Immediate memory**										
Men	−0.139^*^	–	−0.128	−0.211^**^	0.070	0.040	−0.073	−0.021	−0.115^*^	0.086
Women	−0.218^***^	–	−0.042	−0.166^***^	**0.099** ^*^	−0.028	−0.036	0.001	−0.025	0.057
Whole group	−0.195^***^	0.037	−0.061	−0.180^***^	**0.095** ^*^	−0.011	−0.048	−0.003	−0.059	0.068
**Delayed memory**										
Men	−0.117^*^	–	−0.065	−0.152^*^	0.000	0.001	−0.049	−0.068	−0.087	0.043
Women	−0.142^**^	–	0.002	−0.225^***^	**0.096** ^*^	−0.007	−0.004	−0.020	−0.068	0.051
Whole group	−0.136^***^	0.042	−0.016	−0.200^***^	0.061	−0.007	−0.019	−0.037	−0.077^*^	0.054
**Word fluency**										
Men	−0.141^**^	–	−0.054	−0.112	0.087	0.034	0.059	0.017	−0.131^*^	0.051
Women	−0.079	–	0.035	−0.212^***^	**0.119** ^*^	−0.081^*^	0.019	0.008	−0.087^*^	0.050
Whole group	−0.106^**^	−0.104^**^	0.010	−0.188^***^	**0.116** ^**^	−0.040	0.034	0.019	−0.096^**^	0.058
**Digit-symbol test**										
Men	−0.143^**^	–	0.023	0.023	0.078	−0.075	−0.049	0.055	−0.112^*^	0.050
Women	−0.170^***^	–	0.024	−0.103^**^	0.042	−0.105^**^	0.007	−0.032	−0.013	0.049
Whole group	−0.158^***^	−0.169^***^	0.031	−0.078^*^	**0.067** ^*^	−0.095^***^	−0.015	0.005	−0.041	0.076
**Composite score**										
Men	−0.185^***^	–	−0.067	−0.138^*^	0.089	0.021	−0.056	−0.016	−0.151^**^	0.087
Women	−0.184^***^	–	0.014	−0.206^***^	**0.102** ^*^	−0.083^*^	−0.017	0.006	−0.054	0.075
Whole group	−0.189^***^	−0.092^**^	−0.003	−0.197^***^	**0.106** ^**^	−0.054	−0.030	0.003	−0.081^**^	0.084

Data are standardized coefficients, βs, and adjusted coefficients of determination ($R_{\text{C}}^{2}$ for covariates) of the multiple regression models. Significant coefficients are presented in bold characters. Included in the analysis were 334 men and 631 women.

–, variable not introduced in the analysis.

^*^p < 0.05; ^**^p < 0.01; ^***^p < 0.001.

The cultural activities dimension was not significantly associated with any cognitive score. The CR dimensions only explained 3% to 13% of the variance of the cognitive scores, with the exception of the digit symbol test and the overall composite score whose explained variances were substantially higher: 37% and 23%, respectively.

### CR dimensions and MCI

In the Cox regression analyses, education/occupation was significantly associated with a reduced risk of conversion to MCI in men, whereas age, depressive symptoms, ApoE-ε4 alleles, and brain atrophy were significantly associated with an increased risk for both genders (Table 4) and marital status with reduced risk, especially in women.

**Table 4:** Association of cognitive reserve dimensions with conversion to mild cognitive impairment by gender^a^.

		**Cognitive reserve dimensions**				
	**Education/ occupation**	**Midlife cognitive activities**	**Leisure activities**	**Cultural activities**	**Physical activities**
Men	**−1.125** ^*^	0.963	0.118	0.744	**–**0.959
Women	0.232	**–**0.696	**–**0.612	0.721	**–**0.290
Whole group	**–**0.281	**–**0.385	**–**0.251	0.974	**–**0.526
**Covariates**								
	**Age**	**Female gender**	**Marital status**	**Depressive symptoms**	**Trait anxiety**	**Number of drugs**	**ApoE-**ε**4 alleles**	**ApoE-**ε**2 alleles**	**Brain atrophy**
Men	0.036	–	**–**0.084	0.095	**–**0.007	**–**0.023	**1.136** ^**^	0.370	**0.141** ^**^
Women	**0.117** ^**^	–	**−0.585** ^*^	0.108	**–**0.029	0.012	**0.690** ^*^	**–**0.081	**0.133** ^**^
Whole group	**0.073** ^*^	−0.379	**–**0.430	**0.101** ^*^	**–**0.022	0.011	**0.826** ^***^	0.089	**0.139** ^***^

Data are coefficients, βs, of the Cox regression analysis.

^**a**^Included in these analyses were 300 men and 543 women (37 and 57 converters to mild cognitive impairment, respectively) were included in these analyses.

^*^p < 0.05; ^**^p < 0.01; ^***^p < 0.001.

The bold values indicate the significant coefficients.

### CR dimensions and cognitive trajectory

**Figure 3:**
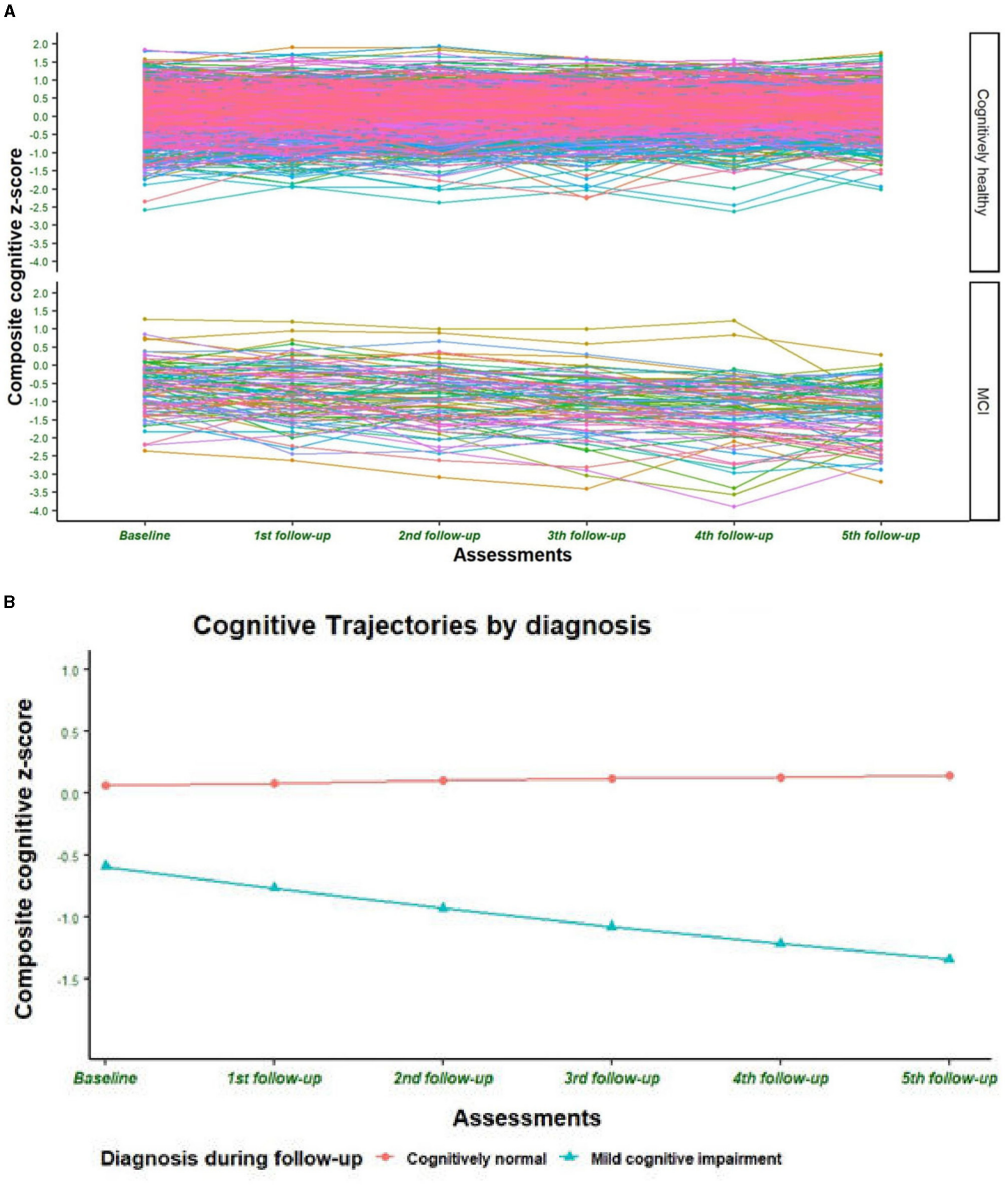
**(A)** Individual cognitive trajectories during follow-up by clinical diagnosis. The figure presents the trajectory of the composite cognitive *z*-score of randomly selected subjects. **(B)** Cognitive trajectory during follow-up by clinical diagnosis. The figure presents the estimated trajectory of the composite cognitive *z*-score according to the first unconditional latent growth curve model where only clinical diagnosis was introduced as an independent factor.

The estimated initial level of the cognitive performance trajectory in CH individuals was *i* = 0.069; the corresponding initial value for individuals with an MCI diagnosis was significantly lower (−0.615, *p* < 0.01). In addition, the linear rate of change of the CH group was positive and significant (*s* = 0.017, *p* < 0.0001) but decreased (−0.149) across time in the MCI group (Figures 3A, B), confirming the research hypothesis and supporting a separate LGCM analysis for the diagnostic group.

**Figure 4:**
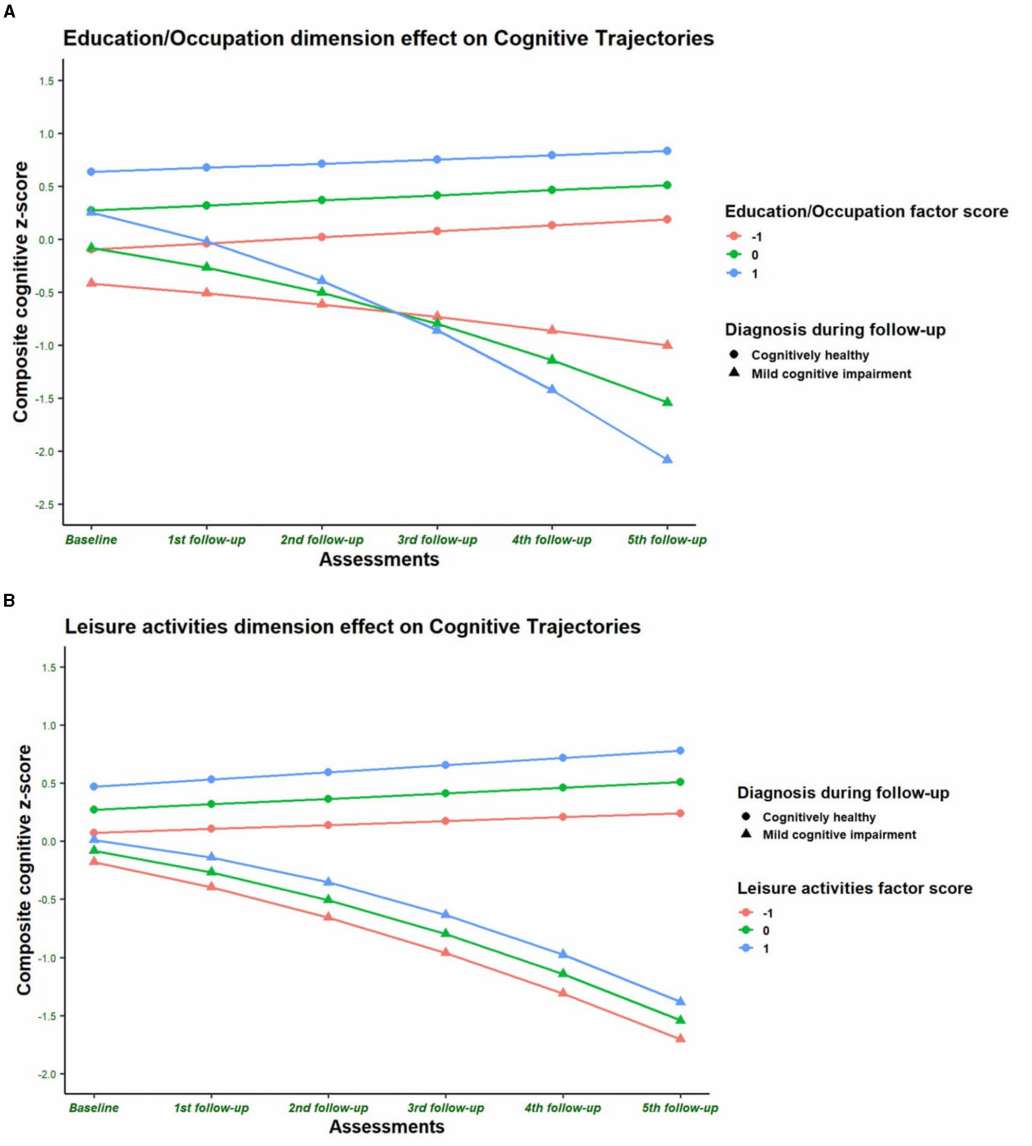
**(A)** Education/Occupation dimension effects on cognitive function across time by clinical diagnosis. **(A)** presents the estimated trajectories of composite cognitive *z*-score in the conditional latent growth curve model of individuals with three different levels of score in the cognitive recognition dimension Education/Occupation. The factor score is significantly and positively associated with the cognitive composite score (CCS) initial level of cognitively healthy subjects (*p* < 0.0001) and for subjects with a diagnosis of mild cognitive impairment (MCI) after follow-up (*p* = 0.045). Education/occupation factor score is also negatively associated with the rate of change of CCS (*p* = 0.015) in subjects with MCI diagnosis. The latter means that the higher the cognitive reserve in terms of education/occupation, the higher the acceleration of the change of rate of CCS in the group of MCI subjects. The conditional latent growth curve model was adjusted by covariates such as age at baseline (centered at 70 years old), gender (0 = men, 1 = women), marital status (0 = married, 1 = other), number of medications, trait anxiety and depression scores at baseline, and number of ApoE-ε4 and ApoE-ε2 alleles. **(B)** Leisure activities dimension effects on cognitive function across time by clinical diagnosis. **(B)** presents the estimated trajectories of composite cognitive *z*-score in the conditional latent growth curve model of individuals with three different levels of score in the cognitive reserve dimension leisure activities. The factor score is significantly and positively associated with the cognitive composite score initial level of cognitively healthy subjects (*p* < 0.0001). The conditional latent growth curve model was adjusted by covariates such as age at baseline (centered at 70 years old), gender (0 = men, 1 = women), marital status (0 = married, 1 = other), number of medications, trait anxiety and depression scores at baseline, and number of ApoE-ε4 and ApoE-ε2 alleles.

The second step was to select the best unconditional LGCM with the right shape of growth. The goodness of fit indices of the linear and the quadratic models were statistically similar for the CH subjects (χ^2^ = 14.958, *df* = 16; Δχ^2^: 6.52, *p* = 0.473), but they were better in the quadratic model (χ^2^ = 10.945, *df* = 12; Δχ^2^ = 27.33, *p* < 0.0001) for the subjects with MCI diagnosis (Table 5a). Therefore, a linear LGCM was performed for CH subjects and a quadratic LGCM for the MCI group with the five CR dimensions as exogenous variables and nine covariates in each model. A positive and significant association was observed between education/occupation and leisure activities with the initial levels of cognitive composite score in both groups (Table 5b and Figures 4A, B), in accordance with the regression analyses of the baseline data. The education/occupation dimension also has a negative and significant association with the rate of cognitive change in the MCI group (Figure 4A). Finally, the intercept is significantly and negatively associated with its slope in the CH group (Table 5b). For the MCI group, the higher the rate of linear change (first year), the lower the rate of quadratic change (latter years of the study) in cognitive function (Table 5b). The complete parameters estimated are shown in Supplementary Table 3.

**Table 5a:** Unconditional latent growth curve model of the composite cognitive score^a^ by final diagnosis.

	**Model fit indices**								**Model comparison tests**		
**Model**	χ^2^	**df**	***p*** **-value**	**CFI**	**TLI**	**RMSEA [CI-95%]**	**RMSEA** ***p*****-value**	**SRMR**	Δ**df**	Δχ^2^	Δ ***p*****-value**
**Cognitively healthy**											
M1. linear	**14.958**	**16**	**0.000**	**1.00**	**1.00**	**0.000 [0, 0,029]**	**1.00**	**0.019**	–	–	–
M2. quadratic	8.438	12	0.001	1.00	1.00	0.000 [0, 0,025]	1.00	0.016	–	–	–
M2 vs. M1									4	6.52	0.473
**Mild cognitive impairment**											
M1. linear	38.276	16	0.001	0.95	0.95	0.119 [0.071, 0.168]	0.013	0.148	–	–	–
M2. quadratic	**10.945**	**12**	**0.001**	**1.00**	**1.00**	**0.000 [0, 0.096]**	**0.726**	**0.04**	–	–	–
M2 vs. M1									4	27.33	0.000

LGCM, latent growth curve model; TLI, Tucker–Lewis index; CFI, comparative fit index; RMSEA, root mean square error of approximation; SRMR, standardized root mean square residual; **χ**^**2**^, chi-square; *df* , degrees of freedom; Δ**χ**^**2**^, chi-square difference; Δ *df* , degrees of freedom difference; Δ *p*-value, *p*-value chi-square difference.

^a^The data of 339 men and 634 women were included in the analyses.

The bold values indicate the significant coefficients.

**Table 5b:** Conditional latent growth curve model of the composite cognitive score^a^ with five cognitive reserve dimensions as exogenous variables.^b^

	**Cognitive healthy**					**Mild cognitive impairment**				
**Growth factors**	**Estimate**	**SE**	**Standardized**	**ci.lower**	**ci.upper**	**Estimate**	**SE**	**Standardized**	**ci.lower**	**ci.upper**
*I*	**0.270** ^***^	0.08	0.471	0.112	0.427	−0.083	0.248	−0.141	−0.568	0.403
*s*	**0.048** ^***^	0.011	0.894	0.027	0.070	−0.157	0.186	−0.524	−0.521	0.208
*q*	**–**	**–**	**–**	**–**	**–**	–0.027^*^	0.011	−0.47	−0.049	−0.006
**Fixed effects**										
Education/Occupation ON i	**0.366** ^***^	0.061	0.410	0.246	0.486	**0.335** ^*^	0.168	0.342	0.007	0.664
Leisure activities ON i	**0.198** ^***^	0.049	0.167	0.101	0.294					
i ON s	–0.056^***^	0.008	−0.594	−0.073	−0.039					
Education/occupation ON q						–0.021^*^	0.009	−0.217	−0.038	−0.004
s ON q						–0.185^***^	0.022	−0.957	−0.228	−0.142
**Random effects**										
i Variance	**0.201** ^***^	0.016	0.616	0.169	0.234	**0.189** ^***^	0.047	0.551	0.097	0.281
s Variance	**0.002** ^***^	**0.000**	**0.557**	**0.001**	**0.002**					
q Variance						**0.00** ^**^	**0.000**	**0.079**	**0.000**	**0.000**

SE, standard error; i, intercept; s, linear slope; q, quadratic slope.

^a^339 men and 634 women were included in the analyses.

^b^Only significant coefficients are presented. All the estimated parameters are presented in the Supplementary Table 3.

^*^p < 0.05; ^**^p < 0.01; ^***^p < 0.001.

The bold values indicate the significant coefficients.

### CR dimensions and brain volumes

The gray matter and total brain volumes were not significantly associated with any CR dimension and were negatively associated with age (β: −0.214 to 0.259, *p* < 0.001) and depressive symptoms (β: −0.091 to .273, *p* < 0.05) in both genders and with the number of medications in men (β: −0.108 and 0.130, respectively; *p* < 0.05; Table 6).

**Table 6:** Association of cognitive reserve dimensions with brain volumes corrected by total intracranial volume by gender.

**Gray matter volume**	**Cognitive reserve dimensions**						
	**Education/ occupation**	**Midlife Cognitive Activities**	**Leisure Activities**	**Cultural Activities**	**Physical Activities**	**Explained variance**	
						$R_{\text{CR}}^{2}$	$R_{\text{M}}^{2}$
Men	0.184	−0.289	0.047	−0.002	0.058	0.003	0.091
Women	−0.032	−0.268	−0.013	0.118	0.093	0.006	0.070
	**Covariates**						
	**Age**	**Marital status**	**Depressive symptoms**	**Trait anxiety**	**Number of drugs**	**ApoE-**ε**4 alleles**	**ApoE-**ε**2 alleles**
Men	**−0.214** ^***^	0.033	**−0.163** ^**^	0.007	**−0.130** ^*^	−0.052	0.023
Women	**−0.281** ^***^	−0.001	**−0.091** ^*^	−0.012	0.018	−0.013	0.036
**Total brain**	**Cognitive reserve dimensions**						
**volume**
	**Education/ occupation**	**Midlife cognitive activities**	**Leisure activities**	**Cultural activities**	**Physical activities**	**Explained variance**	
						$R_{\text{CR}}^{2}$	$R_{\text{M}}^{2}$
Men	−0.013	−0.125	0.011	0.065	0.029	0.011	0.031
Women	−0.027	−0.192	0.007	0.046	0.052	0.002	0.079
	**Covariates**						
	**Age**	**Marital status**	**Depressive symptoms**	**Trait anxiety**	**Number of drugs**	**ApoE-**ε**4 alleles**	**ApoE-**ε**2 alleles**
Men	**−0.258** ^***^	0.018	**−0.276** ^***^	0.114	**−0.108** ^*^	−0.079	0.014
Women	**−0.299** ^***^	0.033	−0.060	0.003	−0.012	−0.005	0.066

Data are standardized coefficients β and adjusted coefficients of determination ($R_{\text{CR}}^{2}$ for cognitive reserve and $R_{\text{M}}^{2}$ for the entire model) of the multiple regression models. Included in the analyses were 334 men and 632 women.

^*^p < 0.05; ^**^p < 0.01; ^***^p < 0.001.

The bold values indicate the significant coefficients.

## Discussion

This study explored the dimensionality of CR and the relative association of every CR dimension with cognitive performance, trajectory, decline, and gross brain structure in a cohort of CH older adult volunteers without major neurological, systemic, or psychiatric conditions. Our hypotheses were partially confirmed: five interrelated dimensions of CR have been identified; three of them, education/occupation, midlife cognitive activities, and leisure activities, are associated with better cognitive performance and provide a buffer against cognitive impairment. A person's education/occupation may delay the clinical onset of MCI and is also associated with the rate of change in cognitive performance in the MCI group. However, these CR dimensions are not related to structural brain volumes.

The multivariate analyses of a set of 21 sociodemographic and lifestyle data, selected in accordance with the conceptual framework of the CR construct, disclosed five dimensions representing the educational, late cognitive, leisure, cultural, and physical activities from childhood to midlife adulthood in this cohort of older adult volunteers. These dimensions are rather similar to other proxy markers arbitrarily selected in previous reports (Harrison et al., 2015; Rouillard et al., 2017), but they have been empirically identified and quantified for every individual with a statistical objective tool and support the assessment of their effect size on the cognitive performance and trajectory of the cohort.

An unsurprising finding is that these five CR dimensions are highly interrelated. Consequently, the strength and relative association of every dimension with cognitive performance and trajectory could be better ascertained if all of them were considered in the statistical models. A second relevant finding is that the relative loadings of the variables presumably related to CR are significantly different between men and women. Long-lasting social and cultural norms and habits determine marked differences by gender in education, occupation, and daily activities that are supposed to generate CR (Hassing, 2020). Moreover, some biological differences in cognitive abilities may also determine a gender-specific CR (Sundermann et al., 2016) and differences in its metabolic and functional neural substrate (Malpetti et al., 2017). Therefore, it would be useful to take into account the gender effect in the analysis of CR.

The education/occupation dimension is significantly associated with almost all the cognitive domains (memory, language, visuo-constructive ability, and executive functioning) in both genders, in accordance with other previous dimensional analyses (Harrison et al., 2015; Rouillard et al., 2017; Feldberg et al., 2021) and its common use as a proxy marker of CR (Stern et al., 2018). The leisure activities dimension is also associated with several cognitive domains, and midlife cognitive activities showed modest additional relevance (Feldberg et al., 2021). Education/occupation and leisure activities strongly explain overall cognition (21% and 24% of the variance of the composite score for men and women, respectively) and seem to represent a lifestyle open to experience and engagement in stimulating actions in early and midlife associated with enhanced cognitive performance level in old age (Ihle et al., 2016). It should be emphasized that all the associations of CR with cognitive performance described in Table 3a have been observed after controlling for the estimated brain atrophy as a proxy of the overall aging effects on the brain (Stern et al., 2018).

Education, professional occupation, and participation in cognitively stimulating activities share an underlying process and are the most relevant factors of CR contributing to late cognitive functioning according to a meta-analysis of 135 studies (Opdebeeck et al., 2016) and other dimensional studies (Rouillard et al., 2017). Our study has simultaneously analyzed five dimensions of CR, verified their high collinearity, and shown that education/occupation (a marker of formal schooling and labor attainment), leisure activities, and midlife cognitive activities (surrogates of informal and cultural interests and abilities) are the main dimensions of CR associated with cognitive performance.

Previous reports (Darby et al., 2017; Lavrencic et al., 2018a; Narbutas et al., 2021) have indicated that CR differentially affects individual cognitive domains. It is significantly associated with attention, executive functions, verbal and working memory, orientation, and semantic knowledge but is not related to emotion perception, processing speed, visuospatial tasks, or motor performance. Our results showed a significant association of some CR dimensions with all the cognitive domains that we have examined. All the cognitive tests selected for our study, which are useful in assessing the cognitive decline and the transition from healthy cognitive aging to MCI (Cherbuin et al., 2010; Mura et al., 2014), as well as the overall composite score, are significantly influenced by the education/occupation dimension. However, the digit symbol test is the most strongly associated with CR dimensions that explain 37% of its variance. This finding suggests that attention, executive functions, memory, and speed of information processing assessed by the digit symbol test (Jaeger, 2018) are most related to CR as a construct. The overall estimate of the impact of CR on cognitive functioning, approximately 30% of explained variance, is in accordance with a recently published study (Martin et al., 2022).

Many previous reports also indicate that age (Lenehan et al., 2015; Li et al., 2017), depressive symptoms (Murphy and O'Leary, 2010; Rock et al., 2014; O'Shea et al., 2015; Lara et al., 2022), number of medications (Cheng et al., 2018; Khezrian et al., 2019), and brain atrophy (Stern et al., 2018) are negatively associated with cognitive performance. In contrast, trait anxiety facilitates performance in several of our cognitive tests, probably because both trait (Salthouse, 2012) and state anxiety (Potvin et al., 2013; Martinussen et al., 2019) have an inverted *U*-shaped relationship with cognitive performance; high levels are harmful but moderate levels may be beneficial.

A recent consensus definition of CR (Stern et al., 2018) suggests that, optimally, CR should moderate the relationship between brain and cognitive changes. However, there is no relevant brain pathology in our cohort, although age, atrophy, and other covariates can be considered surrogates of neural attrition. For this reason, the crossover analyses of the relative influence of every dimension of CR on cognitive performance at baseline are addressed within a reflective model (Jones et al., 2011). Our data indicate that CR explains more variance than these covariates. None of the CR dimensions showed a significant relationship with cognitive trajectory in the group of CH older adults during the follow-up. The latter finding has also been observed in other studies, which indicates that CR has little impact on the cognitive trajectory during aging and does not determine slower cognitive decline in CH individuals (Lane et al., 2017; Soldan et al., 2017; Lavrencic et al., 2018b; Williams et al., 2021), even in the oldest-old individuals (Hakiki et al., 2021), and no less cognitive decline post-retirement in individuals with more complex occupations (Lane et al., 2017). However, these findings should be considered with caution; protective effects on cognitive health and performance might be observed after longer periods of observation (Li et al., 2021) or in the presence of brain pathologies or functional impairment (Ihle et al., 2020). Our data and those of a similar follow-up study (Tucker-Drob et al., 2009) obtained from a cohort of CH people indicate that CR reflects the persistence of earlier differences in cognitive functioning rather than differential rates of age-associated cognitive decline. A recent revision of published data concludes that educational attainment influences late-life cognitive function primarily by contributing to individual differences in cognitive skills that emerge in early adulthood but persist into older age. The authors propose a threshold model to account for the association between educational attainment and late-life cognitive decline and dementia risk (Lövdén et al., 2020). This model is also in accordance with the association of higher CR with a faster decline observed in our group of converters to MCI and in other cohorts of MCI (Soldan et al., 2017; Kato et al., 2022) or individuals with dementia (van Loenhoud et al., 2022).

CR has been considered a relevant factor in delaying the clinical onset of cognitive decline due to neurological disease (Soldan et al., 2017; Stern et al., 2018; Pettigrew and Soldan, 2019; van Loenhoud et al., 2019; Xu et al., 2019). The incidence of MCI is more protracted in elderly people with better estimates of CR (Soldan et al., 2017; van Loenhoud et al., 2019), although CR proxies interact with gray matter volume (van Loenhoud et al., 2019) or ApoE ε4 and ε2 alleles (Pettigrew et al., 2013; Mazzeo et al., 2019; Xu et al., 2019). In this regard, in our cohort, we have also observed a risk reduction associated with the CR dimension education/occupation, but with marked differences by gender; this effect is significant only for men. Several previous studies have found that high education (Tervo et al., 2004; Rapp et al., 2013; Jia et al., 2020; Zhang et al., 2021) is protective against MCI, but other dimensions of CR were not considered, and the effect of gender was usually not reported. Differences in exposure to education and work activities by gender can determine the relevance of this CR dimension in the conversion to MCI (Liu et al., 2021). As reported in many studies, age (Middleton et al., 2007; Hughes et al., 2014; Hamer et al., 2018; Lavrencic et al., 2018b); ApoE-ε4 alleles (Tervo et al., 2004; Kryscio et al., 2006; Luck et al., 2010; Lipnicki et al., 2016); being widowed, divorced, or living alone (Jia et al., 2020); brain atrophy (Jack et al., 2005; van Loenhoud et al., 2019); and depressive symptoms (Gao et al., 2013) are positively associated with conversion to MCI in our cohort. Protective and risk factors for MCI vary by age (Sachdev et al., 2012; Wang et al., 2017) and gender (Lee et al., 2011; Sachdev et al., 2012) and may also interact between them (Hughes et al., 2014). Our data point out that CR dimensions interact with sex and other risk factors for MCI.

Finally, none of the five dimensions of CR built up during childhood and adulthood is significantly associated with baseline brain volumes in this cohort. In accordance with many previous studies, age (Sigurdsson et al., 2011; Dickie et al., 2013), depressive symptoms (Zhang et al., 2020), and the number of medications (Dieleman et al., 2017) also show negative associations. Environmental enrichment and cognitive training have produced increased brain size in experimental animal models (Milgram et al., 2006; Gelfo et al., 2017), but the effects of CR on neural tissue and brain reserve are probably mainly produced by neurogenesis (Steiner et al., 2006; Xu et al., 2015; Saraulli et al., 2017), angiogenesis (He et al., 2017), synaptic density (Ramos-Miguel et al., 2018), or connectivity (Arenaza-Urquijo et al., 2013; Bozzali et al., 2015; Perani et al., 2017; van Balkom et al., 2020; Varela-López et al., 2022). Although global volumetric assessments can hardly capture these tiny structural effects, there are recent reports of higher gray matter volume and metabolism (Arenaza-Urquijo et al., 2013) and increased cortical surface area and thickness (Andrews et al., 2021) in more educated healthy people. However, a recent study indicated that CR modulates cortical architecture only at the pre-dementia stage (Serra et al., 2022). Our study and other recent studies (Valsdóttir et al., 2022) have not been able to detect volumetric changes associated with CR dimensions, perhaps because these measures are not sensitive enough to assess brain reserve at this macroscopic level (Christensen et al., 2009). More precise cortical assessments could disclose positive findings. The studies of the effect of CR on the burden of neurodegenerative lesions are also controversial, showing no relationship (Del Ser et al., 1999; Wolf et al., 2019) or an inverse (Oveisgharan et al., 2020) relationship, probably due to similar methodological limitations and differences.

This study has some limitations. The cohort is not population-based; the selection methods and the exclusion criteria included education level, and the percentage of women was significantly higher than that of the general population of Madrid (https://datos.madrid.es) (Olazarán et al., 2015). The identification of CR dimensions is based on an extensive but limited set of sociodemographic and lifestyle variables that determine the factorial model obtained. Moreover, we have only gross structural and cross-sectional data as surrogates of brain reserve, which may not be sensitive enough to detect the biological substrate of CR probably based on functional connectivity, neural networks, and synaptic density. In addition, the reported associations between CR dimensions and cognitive performances, particularly those of midlife cognitive activities and leisure activities, may be due to reverse causality. We are aware that the relationships between CR, cognitive performance, and intelligence are very complex (Hu et al., 2022). A 5-year follow-up is short when studying the effects of CR on late cognitive abilities that may have been mainly produced during younger ages. Finally, it must be emphasized that our data, as well as other similar studies, do not prove a definite causal relationship between CR proxies and late-life cognitive performance; the alternative explanation that individuals with higher cognitive abilities at a young age are especially involved in the activities related to CR cannot be ruled out.

In summary, our study identified five dimensions of CR derived from the analysis of a set of 21 variables of early and intermediate life activities. These dimensions are highly interrelated, and their structures are similar for men and women, although they have some different components and loadings. Only three of them, education/occupation, midlife cognitive activities, and leisure activities, showed a significant association with cognitive performance late in life, but they were not associated with gross brain volumes. Although these dimensions of CR have no significant relationship with the cognitive trajectories along the 5-year follow-up period, the dimension education/occupation is associated with a risk reduction of MCI for men. These findings not only support the frequent use of education and occupation as proxy markers of CR but also indicate that other dimensions, such as midlife cognitive activities and leisure activities, and gender differences should also be considered. Our findings are also in accordance with the hypothesis that high CR reduces the risk of developing MCI and dementia because it determines better cognitive performance, provides a buffer against the clinical expression of brain disease (Rusmaully et al., 2017), delays the onset of symptoms (Soldan et al., 2017; Lavrencic et al., 2018b), and reduces the effect of neurodegenerative burden (Rusmaully et al., 2017) or the levels of cerebrovascular disease (Del Ser et al., 1999; Pettigrew et al., 2019). CR has no relevant effect on the cognitive trajectory of CH older adults (Soldan et al., 2017; Lavrencic et al., 2018b) but, in contrast, is associated with faster decline in those with MCI (Soldan et al., 2017).

## Data availability statement

The data analyzed in this study is subject to the following licenses/restrictions: Data can be available to any interested researcher upon request. Requests to access these datasets should be directed to TdS, tdelser@fundacioncien.es.

## Ethics statement

The studies involving humans were approved by Ethics Committee of the Carlos III Health Institute, Madrid, Spain. The studies were conducted in accordance with the local legislation and institutional requirements. The participants provided their written informed consent to participate in this study.

## Author contributions

TdS: study concept and design, study supervision, data analysis and interpretation, statistical analysis supervision, and drafting of the manuscript. EV-L: study design, statistical analysis, data analysis and interpretation, and manuscript revision. LJ-E: statistical analysis and interpretation of data and manuscript revision. MÁ-V and LZ: acquisition of neuropsychological data and analysis and interpretation of data and manuscript revision. MV and M-AZ: acquisition of clinical data and analysis and interpretation of data and manuscript revision. MF-B: study design, study supervision, acquisition of neuropsychological data, and analysis and interpretation of data and manuscript revision. All authors contributed to the article and approved the submitted version.
