# Supplementary material for: Dimensions of cognitive reserve and their predictive power of cognitive performance and decline in the elderly
Source: Front Dement. 2023 Aug 31;2:1099059. doi: 10.3389/frdem.2023.1099059 (PMC11285562; doi:10.3389/frdem.2023.1099059)
Supplement: Supplementary file 1 [file Data_Sheet_1.docx]

**Supplementary Table S1. Scheme of statistical analysis according to the research questions and hypotheses posed**

| **RESEARCH QUESTIONS** | **HYPOTHESES** | **ANALYSES**  **(Software used)** | **OBJECTIVES OF THE ANALYSES** |
| --- | --- | --- | --- |
| 1. Is the Cognitive Reserve (CR) structure unidimensional or has several dimensions? | H1: Several different factors are included in the CR construct. | Factorial Analysis (FA)   1. Exploratory FA (R – “psych” version 2.2.5 and functions such as “fa.parallel”, “fa” and "fa.diagram”) 2. Confirmatory FA (R – “lavaan” version 0.6-8)   The sample was randomly divided to conduct a cross-validation procedure. First, exploratory FA was conducted to explore potential factors backed by previous scientific literature abut CR. Then confirmatory FA was conducted to validate the results. | Reduce variables and identify a dimensional structure of CR with good fit indexes^1^. |
| 1.1 Is the CR structure similar in both genders or different? | H1.1: CR internal structure is not invariant for men and women | Multigroup invariance analysis  (R – “lavaan” version 0.6-8) | Assess if the internal structure of CR is invariant across gender. |
| 2.1 Have CR dimensions relevant effects on the cognitive performance of normal individuals in old age? | H2.1: CR dimensions have a positive and significant effect on cognitive performance. | Multiple regression analysis  (SPSS version 20) | Examine the effect of CR dimensions on cognitive performance and the variance explained. |
| 2.2 What is the effect of CR dimensions on cognitive decline in old age? | H2.2: CR dimensions have a positive and significant effect on the conversion to mild cognitive impairment (MCI) | Cox proportional hazards regression analysis  (SPSS version 20) | Assess the effect of CR dimensions on the risk to convert to MCI. |
| 2.3 Are the CR dimensions associated with the trajectory of cognitive functioning in subjects cognitively normal and subjects with diagnosis of MCI during the follow-up?  Before answering the previous question, two  questions must be formulated:  2.3.1 Is the trajectory of cognitive functioning across time different for CH subjects and subjects who will convert to MCI?  2.3.2 What is the shape of the growth curve of cognitive performance in subjects cognitively normal and subjects with diagnosis of MCI at the end of follow-up? Does the trajectory show a linear or nonlinear development? | H2.3: CR dimensions have a positive and significant effect on the level and the rate of change of cognitive performance of subjects.  H2.3.1: The cognitive trajectories (intercept and slope) of subjects with final diagnosis of normal cognition or MCI are significantly different.  H2.3.2: The shape of the growth curve of cognitive performance is different across groups (CH and final MCI) | Conditional LGCM analysis  (R – “lavaan” version 0.6-12, “semTools” version 0.5-6,  “mice” version 3.14-0)  Unconditional LGCM analysis (R – “lavaan” version 0.6.8)  2.3.1 First, a MIMIC^2^ analysis was conducted, to calculate the effect of final diagnosis on growth factors (intercept and slope)  2.3.2 two nested models were separately tested in each group:  1^st^M: Linear model  2^nd^M: Quadratic model | Examine the specificity of the relation between CR dimensions and levels (“intercepts”) and rates of longitudinal change (“slopes”) in cognitive performance  Analyze the effect of final diagnosis on the growth factors.  Compare nested models to select the best model^*^ for each group.  (*) The choice of the model was based on goodness fit^3^, or parsimony when here an equal fit. |
| 3.To what extent CR modifies Brain Reserve? | H3: CR dimensions have a positive and significant effect on brain volume. | Multiple regression analysis  (SPSS version 20) | Examine the effect of CR dimensions on brain volume and the variance explained |

CR: Cognitive reserve; FA: Factorial analysis; MCI: mild cognitive impairment; LGCM: latent growth curve model; ^1^ Goodness of fit indexes were the Tucker-Lewis index (TLI), Comparative fit index (CFI), Root mean square error of approximation (RMSEA), and Standardized Root Mean Square Residual (SRMR)

^2^ Multiple indicators multiple causes

^3^ The overall goodness of fit is indicated by the following indexes: Chi-square difference (Δχ^2), p-value Chi-square difference (Δ pvalue), Tucker-Lewis index (TLI), Comparative fit index (CFI), Root mean square error of approximation (RMSEA), and Standardized root mean square residual (SRMR)

**Supplementary Table S2. Exploratory factorial analysis process**

**Table S2.A. Goodness of fit indices from three different factorial solutions.**

| **Number of factors** | $\boldsymbol{\chi}^{\boldsymbol{2}}$ | $\boldsymbol{df}$ | $\boldsymbol{\Delta\chi}^{\boldsymbol{2}}$ | $\boldsymbol{\Delta df}$ | $\boldsymbol{p(\Delta\chi}^{\boldsymbol{2}}\boldsymbol{)}$ | **TLI** | **RMSEA**  **(IC%90)** | **Average variance explained** |
| --- | --- | --- | --- | --- | --- | --- | --- | --- |
| 4 factors | 277,935 | 132 | -- | -- | -- | 0.899 | 0.059  (0.052-0.065) | 0.433 |
| **5 factors** | **151,244** | **115** | **126,691** | **17** | **0. 000** | **0.932** | **0.049**  **(**0.040-0.056) | **0.465** |
| 6 factors | 105,087 | 99 | 46,157 | 16 | 0. 000 | 0.944 | 0.045  (0.035-0.052) | 0.485 |

*Tucker-Lewis index (TLI), Comparative fit index (CFI), Root mean square error of approximation (RMSEA), Chi square (*$\chi^{2}$*), degrees of freedom (df), Chi square difference (*${\Delta\chi}^{2}$*), degrees of freedom difference (*${\Delta df}^{2}$*)*

**Table S2.B. Standardized factor loadings and communalities of 21 variables and 5 factors.**

|  | **F1** | **F3** | **F2** | **F4** | **F5** | **h2** | **u2** |
| --- | --- | --- | --- | --- | --- | --- | --- |
| Education | **0,67** | 0,22 | 0,07 | 0,00 | -0,09 | 0,63 | 0,37 |
| Languages | **0,31** | 0,20 | 0,02 | 0,13 | -0,15 | 0,24 | 0,76 |
| Education at Adulthood | **0,71** | 0,02 | 0,00 | 0,12 | 0,13 | 0,63 | 0,37 |
| Ocupation | **0,92** | -0,03 | 0,04 | 0,02 | -0,01 | 0,81 | 0,19 |
| Years at work | **0,58** | -0,03 | -0,17 | -0,04 | 0,06 | 0,29 | 0,71 |
| Newspapers | 0,26 | **0,58** | -0,01 | -0,03 | -0,06 | 0,48 | 0,52 |
| Electronic devices | 0,28 | **0,28** | 0,00 | 0,26 | 0,05 | 0,41 | 0,59 |
| Attending shows | -0,02 | **0,64** | 0,05 | 0,10 | 0,14 | 0,54 | 0,46 |
| Music listening | -0,14 | **0,48** | -0,07 | 0,18 | 0,09 | 0,30 | 0,71 |
| Reading | 0,11 | **0,63** | 0,07 | 0,01 | 0,05 | 0,53 | 0,47 |
| Travelling | 0,05 | **0,50** | 0,11 | 0,08 | 0,09 | 0,43 | 0,57 |
| Attending lectures | 0,09 | **0,45** | 0,08 | 0,43 | 0,01 | 0,50 | 0,50 |
| Hobbies | -0,03 | 0,05 | **0,77** | -0,01 | 0,08 | 0,65 | 0,36 |
| Card games | -0,16 | 0,10 | **0,36** | 0,11 | 0,01 | 0,14 | 0,87 |
| Intellectual games | 0,06 | -0,05 | **0,77** | -0,01 | -0,06 | 0,55 | 0,45 |
| Writing | 0,08 | 0,04 | -0,03 | **0,65** | -0,11 | 0,52 | 0,48 |
| Artistics activities | 0,09 | -0,10 | 0,00 | **0,54** | 0,16 | 0,32 | 0,68 |
| Collecting | 0,16 | -0,15 | 0,12 | **0,31** | 0,16 | 0,22 | 0,78 |
| Social Activities | -0,11 | 0,17 | 0,09 | **0,48** | 0,06 | 0,36 | 0,64 |
| Light exercise | 0,04 | 0,23 | -0,04 | -0,04 | **0,60** | 0,41 | 0,60 |
| Moderate exercise | 0,03 | -0,04 | 0,08 | 0,04 | **0,66** | 0,55 | 0,45 |
| SS loadings | 2,83 | 2,48 | 1,52 | 1,79 | 1,13 |  |  |
| Proportion variance | 0,13 | 0,12 | 0,07 | 0,09 | 0,05 |  |  |
| Cumulative variance | 0,13 | 0,25 | 0,33 | 0,41 | 0,46 |  |  |
| Proportion Explained | 0,29 | 0,25 | 0,16 | 0,18 | 0,12 |  |  |
| Cumulative Proportion | 0,29 | 0,24 | 0,70 | 0,88 | 1,00 |  |  |

**Table S2.C. Standardized factor loadings and communalities of twenty-one variables and six factors.**

|  | **F1** | **F4** | **F5** | **F2** | **F3** | **F6** | **h2** | **u2** |
| --- | --- | --- | --- | --- | --- | --- | --- | --- |
| Education | **0.64** | 0.18 | 0.06 | 0.09 | -0.08 | -0.11 | 0.64 | 0.36 |
| Ocupation | **0.90** | -0.05 | 0.04 | 0.03 | 0.02 | -0.01 | 0.82 | 0.18 |
| Education at Adulthood | **0.67** | 0.02 | 0.16 | -0.02 | 0.10 | 0.03 | 0.63 | 0.37 |
| Languages | **0.30** | 0.16 | 0.14 | 0.05 | -0.15 | 0.00 | 0.24 | 0.76 |
| Years at work | **0.59** | 0.02 | -0.15 | -0.07 | 0.03 | 0.13 | 0.30 | 0.70 |
| Attending shows | -0.03 | **0.61** | 0.13 | 0.05 | 0.10 | 0.05 | 0.55 | 0.45 |
| Newspapers | 0.27 | **0.50** | -0.04 | -0.01 | 0.01 | -0.25 | 0.50 | 0.50 |
| Electronic devices | 0.33 | **0.42** | 0.00 | -0.03 | 0.04 | 0.14 | 0.44 | 0.56 |
| Music listening | -0.11 | **0.59** | 0.03 | -0.02 | 0.01 | 0.03 | 0.31 | 0.69 |
| Travelling | 0.10 | **0.56** | 0.03 | 0.05 | 0.06 | 0.16 | 0.48 | 0.52 |
| Reading | 0.04 | **0.53** | 0.15 | 0.06 | 0.11 | -0.19 | 0.54 | 0.46 |
| Attending lectures | 0.07 | 0.18 | **0.51** | 0.08 | 0.04 | -0.02 | 0.51 | 0.49 |
| Writing | 0.08 | -0.05 | **0.73** | -0.04 | -0.03 | 0.02 | 0.53 | 0.47 |
| Social Activities | -0.07 | 0.08 | **0.55** | 0.09 | 0.04 | -0.03 | 0.38 | 0.62 |
| Artistics activities | 0.09 | 0.09 | **0.34** | 0.02 | 0.10 | **0.27** | 0.35 | 0.65 |
| Collecting | 0.23 | 0.09 | 0.17 | 0.04 | -0.03 | **0.32** | 0.28 | 0.72 |
| Intellectual games | 0.09 | -0.05 | 0.00 | **0.74** | -0.04 | -0.02 | 0.55 | 0.45 |
| Hobbies | -0.06 | 0.01 | -0.01 | **0.80** | 0.04 | 0.01 | 0.64 | 0.36 |
| Card games | -0.04 | 0.24 | -0.03 | **0.28** | -0.08 | 0.23 | 0.19 | 0.81 |
| Light exercise | 0.02 | 0.03 | -0.02 | -0.01 | **0.82** | -0.06 | 0.67 | 0.33 |
| Moderate exercise | -0.01 | -0.01 | 0.04 | 0.10 | **0.53** | 0.22 | 0.41 | 0.59 |
| SS loadings | 2.75 | 2.42 | 1.73 | 1.43 | 1.15 | 0.48 |  |  |
| Proportion variance | 0.13 | 0.12 | 0.08 | 0.07 | 0.05 | 0.02 |  |  |
| Cumulative variance | 0.13 | 0.25 | 0.33 | 0.40 | 0.45 | 0.47 |  |  |
| Proportion Explained | 0.28 | 0.24 | 0.17 | 0.14 | 0.12 | 0.05 |  |  |
| Cumulative Proportion | 0.28 | 0.52 | 0.69 | 0.84 | 0.95 | 1.00 |  |  |

**Supplementary Table S3. Model fit comparison for Multigroup invariance test by gender**

|  | $\boldsymbol{\chi}^{\boldsymbol{2}}$ | $\boldsymbol{df}$ | ${\boldsymbol{\Delta}\boldsymbol{\chi}}^{\boldsymbol{2}}$ | $\boldsymbol{\Delta}\boldsymbol{df}$ | ${\boldsymbol{p(}\boldsymbol{\Delta}\boldsymbol{\chi}}^{\boldsymbol{2}}\boldsymbol{)}$ | **TLI** | **CFI** | $\boldsymbol{\Delta}\boldsymbol{CFI}$ |
| --- | --- | --- | --- | --- | --- | --- | --- | --- |
| Configural | 580.88 | 358 |  |  |  | 0.99 | 0.99 |  |
| Weak metric | 1245.51 | 374 |  |  |  | 0.95 | 0.95 |  |
| Weak metric vs Configural |  |  | 112.17 | 16 | <.000*** |  |  | 0.04 |
| Partial Weak Metric 1 | 721.79 | 371 |  |  |  | 0.98 | 0.98 |  |
| Partial WM 1 vs Configural |  |  | 38.76 | 13 | 0.0002*** |  |  | 0.01 |
| Partial Weak Metric 2 | 705.44 | 369 |  |  |  | 0.98 | 0.98 |  |
| Partial WM 2 vs Configural |  |  | 24.25 | 11 | 0.0003*** |  |  | 0.01 |

Chi square ($\boldsymbol{\chi}^{\boldsymbol{2}}$), degrees of freedom (df), Chi-square difference ($\Delta\boldsymbol{\chi}^{\boldsymbol{2}}$), degrees of freedom difference ($\Delta\boldsymbol{df}$), p-value Chi-square difference
${\boldsymbol{p}\boldsymbol{(}\boldsymbol{\Delta}\boldsymbol{\chi}}^{\boldsymbol{2}}\boldsymbol{)}$ , Tucker-Lewis index (TLI), Comparative fit index (CFI), Comparative fit index difference ($\Delta\boldsymbol{CFI}$),

**Supplementary Table S4. Parameters estimated by the conditional LGCM of cognitive composite z-score**

| **Cognitive healthy subjects** | | | | | |
| --- | --- | --- | --- | --- | --- |
| **Paremeters** | **Unstandardized** | **se** | **ci.lower** | **ci.upper** | **Standardized** |
| Mean intercept (i) | 0.27*** | 0,080 | 0,112 | 0,427 | 0,471 |
| Mean linear slope (s) | 0.048*** | 0,011 | 0,027 | 0,070 | 0,894 |
| Education/Occupation ON i | 0.366*** | 0,061 | 0,246 | 0,486 | 0,410 |
| Midlife cognitive act. ON i | 0.076 | 0,099 | -0,118 | 0,271 | 0,079 |
| Leisure activities ON i | 0.198*** | 0,049 | 0,101 | 0,294 | 0,167 |
| Cultural activities ON i | -0.071 | 0,117 | -0,301 | 0,158 | -0,046 |
| Physical activities ON i | -0.017 | 0,059 | -0,132 | 0,098 | -0,015 |
| Gender ON i | -0.083 Ϯ | 0,050 | -0,181 | 0,015 | -0,069 |
| Age at baseline ON i | -0.028*** | 0,006 | -0,040 | -0,016 | -0,187 |
| Depressive symptoms ON i | -0.041*** | 0,012 | -0,064 | -0,018 | -0,160 |
| Trait anxiety ON i | 0.004 Ϯ | 0,003 | -0,001 | 0,010 | 0,075 |
| Number medications ON i | -0.014 | 0,009 | -0,032 | 0,005 | -0,054 |
| APOE e4 alleles ON i | 0.004 | 0,057 | -0,108 | 0,116 | 0,003 |
| APOE e2 alleles ON i | 0.019 | 0,057 | -0,093 | 0,131 | 0,012 |
| Brain trophy ON i | -0.005 | 0,006 | -0,016 | 0,006 | -0,035 |
| Marital status ON i | 0.014 | 0,049 | -0,082 | 0,109 | 0,012 |
| Education/Occupation ON s | -0.009 | 0,009 | -0,027 | 0,010 | -0,103 |
| Midlife cognitive act. ON s | 0.009 | 0,014 | -0,019 | 0,037 | 0,096 |
| Leisure activities ON s | 0.014 Ϯ | 0,007 | 0,000 | 0,028 | 0,125 |
| Cultural activities ON s | -0.009 | 0,017 | -0,042 | 0,025 | -0,058 |
| Physical activities ON s | -0.003 | 0,008 | -0,019 | 0,013 | -0,030 |
| Gender ON s | 0.004 | 0,007 | -0,010 | 0,018 | 0,036 |
| Age at baseline ON s | -0.002 Ϯ | 0,001 | -0,003 | 0,000 | -0,111 |
| Depressive symptoms ON s | 0.005** | 0,002 | 0,002 | 0,009 | 0,223 |
| Trait anxiety ON s | -0.002*** | 0,000 | -0,002 | -0,001 | -0,283 |
| Number medications ON s | -0.001 | 0,001 | -0,004 | 0,002 | -0,044 |
| APOE e4 alleles ON s | -0.021** | 0,008 | -0,036 | -0,006 | -0,147 |
| APOE e2 alleles ON s | 0.000 | 0,008 | -0,015 | 0,016 | 0,002 |
| Brain trophy ON s | -0.001 | 0,001 | -0,003 | 0,000 | -0,089 |
| Marital status ON s | 0.004 | 0,007 | -0,009 | 0,017 | 0,036 |
| i ON s | -0.056*** | 0,008 | -0,073 | -0,039 | -0,594 |

| **Subjects diagnosed of Mild Cognitive Impairment during follow up** | | | | | |
| --- | --- | --- | --- | --- | --- |
| **Paremeters** | **Unstandardized** | **se** | **ci.lower** | **ci.upper** | **Standardized** |
| Mean intercept (i) | -0.083 | 0,248 | -0,568 | 0,403 | -0,141 |
| Mean linear slope (s) | -0.157 | 0,186 | -0,521 | 0,208 | -0,524 |
| Mean quadratic slope (q) | -0.027* | 0,011 | -0,049 | -0,006 | -0,47 |
| Education/Occupation ON i | 0.335* | 0,168 | 0,007 | 0,664 | 0,342 |
| Midlife cognitive act. ON i | 0.062 | 0,263 | -0,454 | 0,579 | 0,062 |
| Leisure activities ON i | 0.095 | 0,169 | -0,236 | 0,426 | 0,078 |
| Cultural activities ON i | 0.179 | 0,318 | -0,445 | 0,803 | 0,117 |
| Physical activities ON i | -0.053 | 0,164 | -0,375 | 0,269 | -0,05 |
| Gender ON i | -0.02 | 0,157 | -0,328 | 0,288 | -0,017 |
| Age at baseline ON i | -0.039* | 0,017 | -0,072 | -0,006 | -0,282 |
| Depressive symptoms ON i | -0.057 Ϯ | 0,031 | -0,118 | 0,004 | -0,21 |
| Trait anxiety ON i | 0.003 | 0,008 | -0,013 | 0,018 | 0,043 |
| Number medications ON i | -0.047 Ϯ | 0,026 | -0,098 | 0,005 | -0,173 |
| APOE e4 alleles ON i | 0.081 | 0,129 | -0,172 | 0,333 | 0,067 |
| APOE e2 alleles ON i | -0.073 | 0,187 | -0,44 | 0,294 | -0,042 |
| Brain trophy ON i | -0.013 | 0,017 | -0,046 | 0,021 | -0,098 |
| Marital status ON i | 0.018 | 0,148 | -0,272 | 0,308 | 0,015 |
| Education/Occupation ON s | -0.07 | 0,176 | -0,415 | 0,275 | -0,139 |
| Midlife cognitive act. ON s | -0.052 | 0,25 | -0,543 | 0,438 | -0,103 |
| Leisure activities ON s | 0.038 | 0,118 | -0,193 | 0,27 | 0,062 |
| Cultural activities ON s | -0.06 | 0,276 | -0,6 | 0,481 | -0,077 |
| Physical activities ON s | -0.024 | 0,117 | -0,254 | 0,206 | -0,044 |
| Gender ON s | -0.133 | 0,121 | -0,369 | 0,104 | -0,217 |
| Age at baseline ON s | 0.017 | 0,015 | -0,014 | 0,047 | 0,234 |
| Depressive symptoms ON s | 0.013 | 0,031 | -0,048 | 0,075 | 0,095 |
| Trait anxiety ON s | -0.008 | 0,006 | -0,02 | 0,003 | -0,266 |
| Number medications ON s | 0.035 | 0,026 | -0,016 | 0,085 | 0,252 |
| APOE e4 alleles ON s | 0.043 | 0,102 | -0,157 | 0,244 | 0,071 |
| APOE e2 alleles ON s | -0.038 | 0,121 | -0,275 | 0,199 | -0,043 |
| Brain trophy ON s | -0.004 | 0,013 | -0,03 | 0,021 | -0,067 |
| Marital status ON s | 0.073 | 0,116 | -0,154 | 0,300 | 0,116 |
| Education/Occupation ON q | -0.021* | 0,009 | -0,038 | -0,004 | -0,217 |
| Midlife cognitive act. ON q | -0.007 | 0,011 | -0,029 | 0,015 | -0,07 |
| Leisure activities ON q | -0.005 | 0,007 | -0,018 | 0,009 | -0,038 |
| Cultural activities ON q | 0.027 Ϯ | 0,014 | -0,001 | 0,055 | 0,18 |
| Physical activities ON q | -0.004 | 0,007 | -0,019 | 0,01 | -0,042 |
| Gender ON q | -0.015* | 0,007 | -0,03 | -0,001 | -0,131 |
| Age at baseline ON q | 0.001 | 0,001 | 0.000 | 0,002 | 0,074 |
| Depressive symptoms ON q | 0.001 | 0,001 | -0,002 | 0,004 | 0,047 |
| Trait anxiety ON q | 0.000 | 0.000 | -0,001 | 0,001 | 0,024 |
| Number medications ON q | 0.000 | 0,001 | -0,003 | 0,002 | -0,004 |
| APOE e4 alleles ON q | 0.000 | 0,006 | -0,011 | 0,012 | 0,001 |
| APOE e2 alleles ON q | 0.018** | 0,007 | 0,005 | 0,031 | 0,106 |
| Brain trophy ON q | -0.001 | 0,001 | -0,002 | 0,001 | -0,058 |
| Marital status ON q | -0.006 | 0,007 | -0,02 | 0,008 | -0,049 |
| i ON s | 0.169 | 0,235 | -0,292 | 0,63 | 0,331 |
| s ON q | -0.185*** | 0,022 | -0,228 | -0,142 | -0,957 |

Ϯ p< 0.10 , * p < 0.05, ** p < 0.01, *** p < 0.001
